# Dr. George H. Briggs

**Published:** 1908-02

**Authors:** 


Dr. George H. Briggs died at Albert Lea, Minn., December 12, 1907, from heart disease aged 80 years. He graduated from the medical department of the University of Buffalo in 1852; served as surgeon of the 8th Wisconsin volunteer infantry during the Civil War; from 1857 to 1859 was superintendent of schools at Delevan, Wis.; and for several years was physician to the Wisconsin Institute for the Deaf and Dumb.

